# Zoonotic and Public Health Implications of *Campylobacter* Species and Squamates (Lizards, Snakes and Amphisbaenians)

**DOI:** 10.3390/pathogens9100799

**Published:** 2020-09-28

**Authors:** Nicodemus M. Masila, Kirstin E. Ross, Michael G. Gardner, Harriet Whiley

**Affiliations:** 1 College of Science and Engineering, Flinders University, GPO Box 2100, Adelaide, SA 5001, Australia; nichodemus.masila@kenttec.go.ke (N.M.M.); Kirstin.ross@flinders.edu.au (K.E.R.); michael.gardner@flinders.edu.au (M.G.G.); 2 Kenya Tsetse and Trypanosomiasis Eradication Council (KENTTEC), P.O. BOX 66290, Westlands, Nairobi 00800, Kenya; 3 Evolutionary Biology Unit, South Australian Museum, North Terrace, Adelaide, SA 5000, Australia

**Keywords:** *Campylobacter* spp., campylobacteriosis, *C. fetus* subsp. *testudinum*, zoonosis, pet squamates, lizard, snake, reptile, One Health

## Abstract

*Campylobacter* spp. is one of the most widespread infectious diseases of veterinary and public health significance. Globally, the incidence of campylobacteriosis has increased over the last decade in both developing and developed countries. Squamates (lizards, snakes and amphisbaenians) are a potential reservoir and source of transmission of campylobacteriosis to humans. This systematic review examined studies from the last 20 years that have reported squamate-associated human campylobacteriosis. It was found that *C. fetus* subsp. *testudinum* and *C. fetus* subsp. *fetus* were the most common species responsible for human campylobacteriosis from a squamate host. The common squamate hosts identified included bearded dragons (*Pogona vitticeps*), green iguana (*Iguana iguana*), western beaked gecko (*Rhynchoedura ornate*) and blotched blue-tongued skink (*Tiliqua nigrolutea*). People with underlying chronic illnesses, the immunocompromised and the elderly were identified as the most vulnerable population. Exposure to pet squamates, wild animals, consumption of reptilian cuisines and cross contamination with untreated water were risk factors associated with *Campylobacter* infections. Proper hand hygiene practices, responsible pet ownership, ‘One Health’ education and awareness on zoonotic diseases will help reduce the public health risks arising from *Campylobacter* exposure through squamates. Continued surveillance using molecular diagnostic methods will also enhance detection and response to squamate-linked campylobacteriosis.

## 1. Introduction

Globally, *Campylobacter* spp. is a common zoonotic pathogen of significant veterinary and public health concern [1,2]. It is the causative agent of campylobacteriosis, a gastrointestinal disease that has been increasing in incidence over the last decade [3,4,5,6]. The disease presents as gastroenteritis with fever, nausea, vomiting, abdominal pains and watery or bloody diarrhea [7]. While the disease may generally be a self-limiting enterocolitis, clearing on its own within a week, it may also manifest in serious long-term complications including extra-intestinal infections and autoimmune disorders such as Guillain-Barré syndrome, Miller-Fisher syndrome, cholecystitis, inflammatory bowel syndrome and reactive arthritis [7,8,9]. Over the last decade the incidence of campylobacteriosis has increased in both developed and developing countries [2]. In the USA, it is estimated that *Campylobacter* spp. causes over 1.3 million cases and approximately 130 deaths per year, with the Foodborne Diseases Active Surveillance Network (FoodNet) reporting an increase in annual incidence rate of human campylobacteriosis from 14.3 in 2012 to 19.5 cases per 100,000 population in 2019 [2,10,11,12,13]

*Campylobacter spp.* presents a threat to human and animal health because of its zoonotic potential, wide host range, ability to colonize diverse habitats, and emerging resistance to some of the commonly used antimicrobial drugs [14]. The virulence of different *Campylobacter* species and severity of the resulting enteritis is dependent on the pathogenesis mechanisms used, including adhesion to the intestinal wall, colonization of digestive tract, invasion of targeted cells and toxin production [15]. The infection process involves penetration of the gastrointestinal mucus by the bacteria using its high motility and spiral shape, adherence to the gut enterocytes and then inducing diarrhea through release of toxins mainly enterotoxins and cytotoxins [16]. While *Campylobacter jejuni* is a fastidious bacterial pathogen, its virulence is adversely affected by environmental stresses such as nutrient insufficiency, heat stress, absence of water, partial oxygen tension above 10%, low PH, UVB exposure and hydrostatic pressure [17]. However, it is able to develop survival mechanisms which include; persisting in the environment, especially in water, in a viable but non-culturable state [18], transition from rod to coccoid shape [19] and growth in biofilm [20]. By altering gene expression pathways, *C. jejuni* can also adapt to new growth temperatures when exposed to a sudden temperature upshift [21] and persist and grow intracellularly in non-phagocytic host cells through the use of gene encoding catalase (katA) enzyme [22]. While previous studies have provided useful information on virulence of *Campylobacter* spp., further research is needed to inform interpretation of different virulence associated markers or genes.

The *Campylobacter* genus displays wide taxonomic diversity currently comprising of 32 species and 9 subspecies [23]. *Campylobacter* spp. is responsible for 9% of all foodborne illnesses in the United States [10] and molecular typing techniques suggest that up to 80% of human infections are caused by *Campylobacter* strains associated with poultry hosts [24]. *Campylobacter jejuni* is the most common *campylobacter* species isolated from human cases with campylobacteriosis [2,25,26]. Additionally, *C. jejuni* causes over 80% of human campylobacteriosis cases, with 50–80% of the cases attributed to the chicken reservoir (both broilers and laying hens) [27,28].

The disease is not only a food-borne illness but is also transmitted through environmental reservoirs including animals [29,30]. Changes in land use, habitat loss, urbanization, encroachment of people into wildlife habitats and community composition are reported to influence wildlife health [31]. With human–wildlife interactions becoming more common, the likelihood of zoonotic spread of campylobacteriosis is increasing [28,32,33]. However, information about horizontal transmission of *Campylobacter* through non-foodborne routes is limited, and the zoonotic nature of the disease is often overlooked [32,34]. One potentially overlooked host is squamates [22]. *Squamata* is the largest order of reptiles comprising of three suborders: lizards (suborder: *Lacertilia/Sauria*), snakes (suborder: *Serpentes/Ophidia*) and worm lizards (suborder: *Amphisbaenia*) [35]. The suborder lizards includes skinks (family: *Scincidae*), dragons (family: *Agamidae*), monitor lizards/goannas (family: *Varanidae*), geckos (family: *Gekkonidae*) and flat-footed lizards (family: *Pygopodidae*) which are all adapted to diverse environments [35]. The squamates have been implicated in potentially aiding horizontal transmission of *Campylobacter* spp. either by cross-contamination through their feces, pet handling or generally as a result of close interaction with human habitats [34].

With the propensity to keep reptiles, including squamates, as pets increase globally [36,37,38], zoonotic disease transfer to humans continues to pose a serious challenge to the public and environmental health sector. This review examines the literature pertaining to squamate-linked campylobacteriosis in humans. Studies describing human campylobacteriosis cases linked to the handling of captive and wild squamates or cross-contamination through their feces are surveyed. Further, trends in emerging *Campylobacter* subspecies, the lizard and snake species involved in transmission and possible exposure routes were also explored. This information will inform more effective management strategies to reduce the risk of zoonotic transfer of *Campylobacter* from captive and wild squamates to humans.

## 2. Results

**Figure 1:**
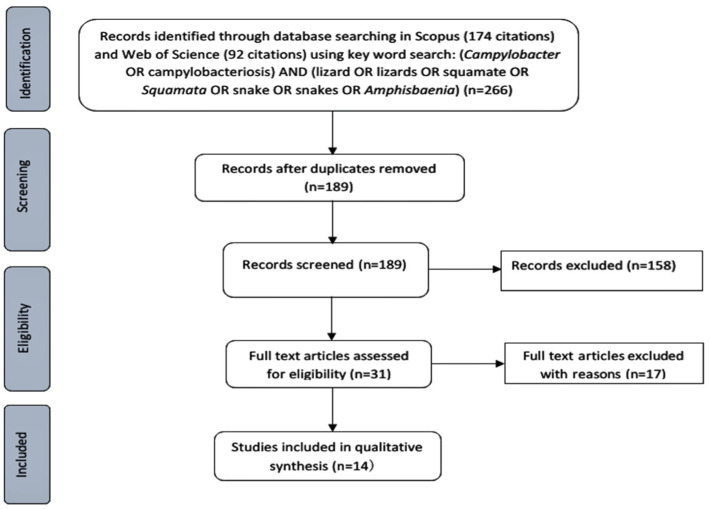
Flow diagram of search methods and articles’ inclusion and exclusion criteria.

One hundred and eighty-nine papers were retrieved from SCOPUS and Web of Science using the search terms identified (Figure 1). After applying the inclusion and exclusion criteria described in Figure 1, a total of 14 papers were included for review; six case studies investigated the source of human campylobacteriosis cases linking them to a squamate source via testing, and eight environmental surveillance studies which screened different squamates for *Campylobacter.*

Table 1 provides a summarized list of squamate species identified from all studies included in this review and the associated *Campylobacter* species which they have been shown to transmit to humans. *Campylobacter fetus* subsp. *fetus* and *C. fetus* subsp. *testudinum* were identified as the most frequently isolated species in reptiles and the predominate causes of human campylobacteriosis linked to squamates. *C. jejuni* and *C. iguaniorum* have also been frequently isolated in squamates and reported to pose a potential health risk to humans. However, no human *C. iguaniorum* infections have been reported yet. [39]. The common lizard hosts identified included bearded dragons (*Pogona vitticeps*) [34,40], western beaked gecko *(Rhynchoedura odura)* [34], *Hydrosaurus pustulatus* [41], green iguana *(Iguana iguana*) [42], *Pogona henrilawsonii, Sauromalus ater, Hemitheconyx caudicinctus* [43] and blotched blue-tongued skink *(Tiliqua nigrolutea)*[44,45]. Five snake species were also identified namely; *Heterodon nasicus*, *Orthriophis taeniurus, Boa constrictor, Python reticulatus* [45] and *Morelia amethistina* [32].

The squamate surveillance studies (Table 2) and case reports (Table 3) identified in this review included reports of squamates contamination with *Campylobacter* from Australia [34], Korea [46], Taiwan [32,41], USA [47,48,49] China [50,51], United Kingdom [44] and Netherlands [39,40,42,43].

In the studies analyzed for this review, molecular methods had been widely used to identify *Campylobacter spp.* isolates and analyze their epidemiology and population genetics. For example, in a reptile surveillance study by Wang et al. [32] involving 179 reptile fecal samples, 16S rRNA sequencing and biochemical methods were used to identify the positive *Campylobacter fetus* species. Using published subspecies-specific sequences and genomic data retrieved from GenBank and MLST database (http://pubmlst.org/), multiplex PCR was used to identify *C. fetus* subsp. *fetus* among the positive samples. Multilocus sequence typing (MLST) was then used to genotype the isolates and analyze for population genetics. Additionally, in a comparative genomics study by Gilbert et al. [39], *C. fetus* subsp. *testudinum* and *C. iguaniorum* isolates from reptiles’ (lizards, chelonians and snakes) fecal samples and human blood samples, were genomically compared with two strains of *Campylobacter iguaniorum (Cig)* isolated from lizards, *Pogona vitticeps* (*Cig* 1485E) and *Iguana iguana* (*Cig* 2463D). For all strains used in the study [39], comparison was done based on 16S rRNA, *atp*A gene sequences, MLST and matrix-assisted laser desorption/ionization-time of flight mass spectrometry (MALDI-TOF MS) to determine genotypic and phenotypic characters of the pathogens. The use of molecular methods such as whole genome sequencing (WGS) inform a better understanding of host adaptation, phylogeny and evolution of emerging *Campylobacter* strains differentiating them from recognized sub-species of *C. fetus*. The studies also confirmed zoonotic potential of *Campylobacter* spp. associated with squamate hosts.

The non-food related risk factors associated with *Campylobacter* infections include handling of pet squamates and wild animals, cross-contamination with surface waters contaminated by wild animals or through contact with lizard feces [34,52]. Where squamates are reared for pet trade or as companion animals, transmission may occur through cross-contamination or contact with their feces when cleaning their vivaria. In communities where reptiles are reared for food, consumption of reptile cuisines predisposes humans to *Campylobacter* infections [50]. People with chronic underlying illnesses, the elderly and the immunocompromised [46,47,49,51], were identified as the most vulnerable population associated with *Campylobacter* infections linked to both consumption of reptilian cuisines and human–pet contact practices. The common clinical signs of human campylobacteriosis associated with a reptilian source included productive cough, fever, epigastric pain, diarrhea and general body weakness [46,47,48].

## 3. Discussion

Reptiles kept as pets offer aesthetic, economic and cultural value in many parts of the world both historically and currently in traditional and modern societies [53]. There are also reported mental and physical benefits which people derive from pet ownership and companionship [54]. However, the increasing popularity of exotic pets, including lizards, snakes and turtles [36,37,38,55], coupled with the emerging novel strains of *Campylobacter* in squamates warrants serious concern from public health practitioners as the pets may harbor diseases or aid in transmission of pathogens of zoonotic potential. An estimated 60% of all known infectious disease pathogens and up to 75% of emerging infectious diseases are zoonotic and able to infect other host species [56,57]. Direct and indirect contact of people with domesticated squamates coupled with failure to adhere to proper hand hygiene and pet care practices potentially presents a risk of transmitting *Campylobacter* spp., especially *C. jejuni* and variants of *Campylobacter fetus* to humans [41,47]. This review presents evidence that squamate-associated campylobacteriosis is a potential public health threat globally.

*Campylobacter fetus* is an opportunistic zoonotic species that poses public health risks to immunocompromised people, patients with underlying chronic illnesses, young children, pregnant women and the elderly [58]. One of its subspecies, *C. fetus* subsp. *fetus* has a wide host range in vertebrate hosts and has veterinary significance as it causes abortion in cattle and sheep. Additionally, *C. fetus* subsp. *venerealis* is host restricted and causes high economic losses in cattle through infertility, abortions and lowered pregnancy rates. The subspecies has also been isolated from blood samples in humans presenting with bacteremia, infective aneurysm and vaginosis [59]. Lastly, the zoonotic *C. fetus* subsp. *testudinum* strain that is primarily found in healthy reptiles, and also in ill snakes, is transmissible and pathogenic to humans [39,49,60,61]. However, despite high incidences of *C. fetus* infections from the three species, bovine genital campylobacteriosis is the only OIE notifiable disease from the *Campylobacter* genus requiring mandatory reporting to the World Organization for Animal Health (Office International des Epizooties—OIE) [62]. There is need for increased knowledge and education on the zoonotic and public health risks of campylobacteriosis at the human–animal-environment interface. Additionally, appropriate approaches need to be implemented to manage emerging zoonotic strains such as *C. fetus* subsp. *testudinum* which continue to remain underestimated in humans, as evidenced by studies analyzed in this review.

One example of a collaborative approach applicable to managing zoonotic infections is the One Health concept. One Health is a multidisciplinary and holistic concept that recognizes interconnections of different components of ecological communities and the inextricable link between human, animal and environmental health through interfaces with food, livestock, wildlife and pathogens in the environment [63]. Human campylobacteriosis associated with squamates’ exposure is thus a One Health issue due to its relevance to food safety, zoonoses and antimicrobial resistance; which are health threats addressed by World Health Organization (WHO), OIE and the Food and Agricultural Organization (FAO) [64,65,66]. In this regard, implementation of a coordinated One Health approach would foster interdisciplinary collaboration, communication and sharing of resources to develop effective surveillance techniques, molecular diagnostic and therapeutic interventions that enhance health outcomes at the human–wildlife–livestock-environment interface. A One Health Zoonotic Disease Prioritization tool bringing together experts from human, animal, wildlife and environment health sectors to prioritize endemic and emerging zoonoses of greatest national concern in a country/region was developed by the Centers for Disease Control and Prevention (CDC) and successfully utilized in prioritizing zoonoses in seven countries [67]. The One Health approach was also successfully applied in the UK through multi-agency coordination, improved biosecurity, surveillance and public health programs leading to decline of human *Salmonella* infections in the 1990s [68,69]. The approach may therefore also find relevance and application in campylobacteriosis prevention, detection and response. The OIE Wildlife working group continues to provide appropriate guidelines that address increasing risk of disease spill over from wildlife to humans and domestic animals through capture, handling, poorly regulated trade and consumption of wildlife.

Squamates can also play a role in cross contamination of other environmental sources of human campylobacteriosis [29,52]. This is particularly a concern with captive squamates which have increased interaction with the built environment. There is also a higher pathogen carriage rate and shedding in captive lizards compared with free-living wild lizards. For example, a Malaysian study by Cheng, Wong and Dykes [70] found 83.3% of captive pet lizards were positive for *Salmonella* while only 25% of free-living wild lizards tested positive. Stressed animals are also more likely to shed more pathogens [71]. Stress could be attributed to abiotic environmental challenges and confinement-specific stressors that contribute to reduced fitness of a captive animal [72], thus there is need for animal welfare concerns to be addressed so as to avoid stress levels that may lead to shedding of disease pathogens by household pet squamates and animals in petting zoos. Although research has focused more on transmission of zoonoses from farm animals to humans, household pets and animals in petting zoos have also been identified as potential sources of exposure to campylobacteriosis for people who may typically not live on or visit farms but have contact with these captive animals [73,74].

Pet care education and responsible pet ownership is crucial in addressing physiological stress on captive exotic pets through ensuring proper housing/enclosures, diet, cleanliness and hygiene, temperature, UV light, humidity control and veterinary health care [75,76]. A study by Vučinić et al. [77] on reptile ownership, demographics and reliance on veterinary care in Balkan countries noted that 40% of pet reptile owners had never contacted veterinarians about medical conditions of their pets. Reptiles pose a significant zoonotic risk to pet owners, zookeepers and veterinarians as well as to the immunocompromised, young children and the elderly [78]. Sensitization on pet-associated disease risks, adherence to proper hygiene and human–animal contact practices thus need to be upscaled.

Reptiles, particularly lizards and snakes, are the primary reservoirs of the emerging *Campylobacter* species. One of these subspecies is the novel *Campylobacter iguaniorum* strain which is closely related to *C*. *fetus* subsp. *testudinum* and both colonize the same reptilian hosts [39]. In a study by Gilbert et al. [39], *C. iguaniorum* isolated from reptilian hosts (*Pogona vitticeps* and *Iguana iguana*) was compared with genomes of closely related reptilian *C. fetus* clade (*C. fetus, C. hyointestinalis and C. lanienae*). Homology was highest between *C. iguaniorum* and reptilian *C. hyointestinalis* and *C. fetus* than between *C. iguaniorum and* mammalian *C. fetus* strains. This may explain the possibility of lateral gene transfer as a result of sharing same host. Other reptiles species such as turtles and tortoises that are phylogenetically distant from squamates [79], are also increasingly popular as pets in some European and Asian countries [80]. Freshwater turtles are farmed in China for human consumption, consequently posing reptilian-associated *Campylobacter* infection risks to humans [81] in situations where food safety and proper hygiene practices are not adhered to.

As shown in Table 1, *C. iguaniorum, C. fetus* subsp. *fetus* and *C. fetus* subsp. *testudinum* were the most common subspecies of *Campylobacter* associated with the ectothermic squamates. Although *Campylobacter jejuni* has also been isolated in lizards, it is typically found in mammals and birds, which are endotherms. This differential distribution could be explained by the optimal temperatures for growth in endotherms and ectotherms. The temperature range of ectothermic vertebrates is 5–46 °C while the optimal temperature for growth in *C. iguaniorum* and *C. fetus subsp. testudinum* is 20–37 °C [82]. Since the mean voluntary temperature for reptiles ranges between 20–35 °C, this temperature range may be an adaptation favoring growth of the pathogens in the reptilian host [40]. On the other hand, with mammals and birds having constant body temperature of 37 and 41–42 °C, respectively, these temperatures would favor the growth of thermophilic *C. jejuni* whose optimal growth temperature is 37–42 °C [17].

## 4. Materials and Methods

This systematic literature review is based on an adapted version of the PRISMA statement [83]. A systematic search of the databases SCOPUS and Web of Science was performed using the search strategy detailed in Figure 1. Briefly, articles written in English over the last 20 years, with the key words; “(*Campylobacter* OR campylobacteriosis) AND (lizard OR lizards OR snake OR *Squamata* OR squamate OR snakes OR *Amphisbaenia*)” were searched for in Scopus (Elsevier, Netherlands) (n = 174) and in Web of Science (Web of Science core collection, Clarivate analytics, United States) databases (n = 92). In Web of Science, the following Boolean search string was used; “TS=(*Campylobacter* OR campylobacteriosis) AND TS = (lizard* OR Lacertilia OR Serpentes OR snake* OR amphisbaenian* OR squamate OR *Squamata* OR Bipedidae OR Blanidae OR Cadeidae OR Rhineuridae OR Trogonophidae OR Dibamidae OR Gecko* OR Pygopodidae OR Agamidae OR Agamas OR dragon* OR Chamaeleonidae OR Chameleon* OR Corytophanidae OR basilisk OR Crotaphytidae OR Hoplocercidae OR clubtail* OR Iguanidae OR Iguana* OR Leiosauridae OR Liolaemidae OR swifts OR Opluridae OR Phrynosomatidae OR Polychrotidae OR Tropiduridae OR Alopoglossidae OR Gymnophthalmidae OR Lacertidae OR Teiidae OR Anguidae OR slowworm* OR Anniellidae OR Helodermatidae OR “gila monster*” OR Xenosauridae OR Lanthanotidae OR Shinisauridae OR Varanidae OR “monitor lizard*” OR Acrochordidae OR Aniliidae OR Anomochilidae OR Boidae OR boa* OR Bolyeriidae OR Colubridae OR colubrid* OR Cylindrophiidae OR Elapidae OR cobra* OR mamba* OR krait* OR elapid* OR asp* OR Homalopsidae OR Lamprophiidae OR Loxocemidae OR Pareatidae OR Tropidophiidae OR python* OR Uropeltidae OR viper* OR pitviper* OR rattlesnake* OR Xenodermatidae OR Xenopeltidae OR Gerrhopilidae OR Leptotyphlopidae OR Typhlopidae OR Xenotyphlopidae) AND TS = (((Public OR human) NEAR/2 (health OR disease OR contaminant*)))”.

Once duplicates were removed (n = 189), titles and abstracts of the results obtained were read and initially excluded if they were review articles, or did not refer to human campylobacteriosis or *Campylobacter* spp. infection in humans (n = 31). Articles were then read in full and excluded if they referred to non-squamate transmitted campylobacteriosis, or if they referred to a squamate-linked *Campylobacter* spp. infection that had not been confirmed through animal testing. Articles were included if they referred to squamate-transmitted campylobacteriosis confirmed through testing of the animal or comparing the animal and human *Campylobacter spp.* isolates. Environmental surveillance studies investigating squamates as a potential risk for human campylobacteriosis were also included.

## 5. Conclusions

There has been increasing popularity of pet squamates globally as well as rising incidence of campylobacteriosis over the last decade. This review provides evidence that squamates may harbor *Campylobacter* spp. and are able to transfer them to humans through contaminated food and water, pet handling or cross-contamination through their feces. Improved educational efforts especially in ‘One Health’ as an emerging approach recognizing the inextricable link between human, animal and environmental health, will help in ensuring the general public, farmers and pet reptile owners are aware of the potential risks and zoonotic implications of campylobacteriosis from pet lizards and snakes. Knowledge and awareness about zoonotic diseases should be enhanced through harmonized and collaborative approaches among human, veterinary, and public health personnel. Additionally, there is need for proper adherence to hand hygiene, pet care services and improved human–animal contact practices in homes and petting zoos. Lastly, continued surveillance of emerging *Campylobacter* species through use of laboratory diagnostic tools and modern molecular techniques will aid in detection that informs more effective management strategies, hence leading to improved public health outcomes.

## Figures and Tables

**Table 1:** Summary of squamate species and the respective *Campylobacter* spp. of human health significance that they have been shown to carry.

Squamate Species	*Campylobacter* spp.	Sequence Data	Reference
Lizard (*Pogona vitticeps*)	*C. iguaniorum*	Whole Genome Sequencing (WGS)	[40]
Lizard (*Iguana iguana*)	*C. iguaniorum* subsp. *nov*	WGS	[42]
Snake (*Heterodon nasicus*)	*C. fetus* subsp. *testudinum*	WGS	[45]
Lizard (*Tiliqua nigrolutea*)	*C. fetus*	Multilocus sequence typing (MLST), PCR	[44]
Lizard (*Pogona vitticeps*)	*C. jejuni*	Quantitative PCR (qPCR)	[34]
Lizard (*Rhynchoedura ornate*)	*C. jejuni*	qPCR	[34]
Lizard (*Hydrosaurus pustulatus*)	*C. fetus* subsp. *testudinum pet-3*	WGS	[41]
Lizard (*Pogona henrilawsonii*)	*C. iguaniorum*	MLST	[43]
Snake (*Morelia amethistina*)	*C. fetus* subsp. *fetus*	Multiplex PCR, MLST	[32]
Lizard (*Hydrosaurus pustulatus*)	*C. fetus* subsp. *fetus*	Multiplex PCR, MLST	[32]
Lizard (*Sauromalus ater*)	*C. iguaniorum*	MLST	[43]
Lizard (*Hemitheconyx caudicinctus*)	*C. iguaniorum*	MLST	[43]
Snake (*Python reticulatus*)	*C. fetus* subsp. *testudinum*	MLST	[45]
Lizard (*Tiliqua rugosa*)	*C. fetus* subsp. *testudinum*	MLST	[45]
Snake (*Boa constrictor*)	*C. fetus* subsp. *testudinum*	MLST	[45]
Snake (*Orthriophis taeniurus*)	*C. fetus* subsp. *testudinum*	MLST	[45]
Lizard (*Tiliqua nigrolutea*)	*C. fetus* subsp. *testudinum*	MLST	[45]

**Table 2:** Surveillance studies investigating squamates as a potential risk for human campylobacteriosis.

Country	Findings	*Campylobacter* spp.	Squamate	Comments	Reference
Taiwan	179 reptile fecal samples obtained from chelonians, lizards and snakes. 12/179 (6.7%) were positive for *Campylobacter* spp.; 10/103 (9.7%) chelonians; 1/56 (1.7%) lizards and 1/20 (5%) of snakes were positive for *C. fetus* subsp. *fetus.*	*C. fetus* subsp. *fetus*	Captive and wild lizards and snakes	Only the captive reptiles’ fecal samples tested positive for *C. fetus.* There were no positive isolates from the 23 reptiles collected from the wild fields.	[32]
Taiwan	Complete genome sequence of *C*. *fetus* subsp. *testudinum* strain pet-3 was isolated from a lizard	C. *fetus* subsp. *testudinum* strain pet-3	Lizard (*Hydrosaurus pustulatus*)	Isolated from humans, lizards, and turtles	[41]
USA	Polyphasic study to determine taxonomy of 13 *C. fetus*-like strains using MALDI-TOF MS yielded a novel *Campylobacter fetus* subsp. *testudinum* subsp. *nov*.	Five reptile *C. fetus*-like strains and eight *C. fetus* strains isolated from humans	Five reptiles	The 13 strains are closely related to *C. fetus* and they had multiple phenotypic biomarkers differentiating them from known *C. fetus* subspecies	[49]
Netherlands	*C. iguaniorum* is genetically related but distinct from *C. fetus* and *C. hyointestinalis*	*C. iguaniorum*	Bearded dragon (*Pogona vitticeps)*	*C. iguaniorum* isolated from a lizard. First whole genome sequence of *C. iguaniorum* was established.	[40]
Australia	33% (17/51) of lizards’ feces collected from central Australia tested positive for *C. jejuni* by quantitative PCR	*Campylobacter jejuni*	46 wild lizards (unknown); five captive lizards (*Pogona vitticeps* and *Rhynchoedura ornate)*	3/5 (60%) of captive lizards; 14/46 (30%) wild lizard fecal samples were positive for *C. jejuni.*	[34]
Netherlands	Initial PCR and 16S rRNA showed the pathogens were most closely related to *C. fetus* and *C. hyointestinalis*. However, a polyphasic study involving characterization by 16S rRNA, atpA and MALDI-TOF MS showed divergence from all other known *Campylobacter* species.	*C. iguaniorum* subsp. *nov*	Five strains isolated from lizards and chelonians	Pathogen isolated from reptiles. Growth of the strains at ambient temperature may be an adaptation to their reptilian hosts which are identified as lizards and chelonians.	[42]
Netherlands	*Campylobacter* spp. through PCR as follows; 38% (62/163) in lizards, 32% (32/100) in snakes. Using culture; 3% (3/100) in snakes, and in 11% (18/163) lizards.	*C. iguaniorum, C*. *fetus* subsp. *testudinum* and *C. hyointestinalis*	Lizards (*Pogona henrilawsonii, Sauromalus ater, Hemitheconyx caudicinctus)* and snakes.	Lizards and snakes carry one or more of the intestinal epsilonproteobacteria. Presence of intestinal *Campylobacter* spp. was higher in lizards than in snakes.	[43]
Netherlands	Despite sharing the same host, no recent recombination was detected when genome comparison of *C. iguaniorum* and closely related *C. fetus* was done. Homology was higher between *C. iguaniorum* and *C. fetus* subsp. *testudinum* than between *C. iguaniorum* and mammalian *C. fetus* (*C. fetus* subsp. *fetus* & *C. fetus* subsp. *venerealis).*	*C. iguaniorum*	Bearded dragon (*Pogona vitticeps*) and green iguana (*Iguana iguana*)	Primary reservoir reported to be reptiles, chelonians and lizards. *C. iguaniorum* strain 1485E and 2463D isolated from bearded dragon and green iguana respectively were genomically compared with reptilian *C. fetus* subsp. *testudinum*.	[39]

**Table 3:** Case studies investigating *Campylobacter* in squamates and links to human campylobacteriosis.

Country	Findings	*Campylobacter* spp.	Squamate	Comments	Demographics	Reference
UK	Four isolates from ill patients were confirmed as reptile *C. fetus* strains using sap insertion PCR. Both strains (mammalian *C. fetus and* reptile *C. fetus)* were characterized by multilocus sequence typing to be sharing 92% nucleotide sequence identity.	Reptile *C. fetus* and classical mammalian *C. fetus (C. fetus* subsp. *fetus and C. fetus* subsp. *venerealis)*	One snake (*Heterodon nasicus*) and one blotched blue-tongued skink *(Tiliqua nigrolutea*)	Reptile-like *C. fetus* strains have been isolated from cases of human disease. They showed capability of infecting humans despite having separate genomospecies. There was evidence of recombination.	Isolates from six clinically ill patients confirmed as reptile *C. fetus* strains using *sap* insertion PCR.	[44]
USA	Two *Campylobacter* spp. with markers of reptile origin were isolated from blood sample of a patient who was symptomatic due to recurrent bacteremia caused by *C. fetus subsp. fetus.* The second isolate was found 37 days after antibiotic therapy	*Campylobacter fetus*	Reptilian origin. Not reported how the patient acquired the pathogen. Chelonian cuisine or contact with pet reptile was suggested.	Pathogen was not able to be identified phenotypically at first. Molecular analysis (16S rRNA, then PCR, *Sap*D sequencing) confirmed the pathogen was similar to *C. fetus* subsp. *fetus* and was of reptilian origin.	A febrile 27-year-old patient with precursor T-cell acute lymphoblastic leukemia.	[48]
China	Identification by multilocus sequence typing (MLST) 13 human cases of *Campylobacter* infection reported in Guangzhou in 2012 to 2013	*Campylobacter fetus* subsp. *testudinum*	Reptilian origin; Food or human–squamate contact was reported as most likely source as reptiles formed an integral part of Chinese cuisine.	Epidemiological data was unavailable for these nine cases.	13 human cases of *C. fetus* reported.	[50]
Korea	Infectious spondylitis with bacteremia in a patient with chronic kidney disease was detected through 16S rRNA gene sequencing	*C. fetus* subsp. *testudinum*	Reptile	*C*. *fetus* spondylitis is a very rare disease. Confirmation of the identity of the squamate linked to the transmission was lacking.	83-year-old male patient with end stage renal disease.	[46]
China	*C. fetus* subsp. *testudinum* strain 772 isolated from the ascites of a patient. Whole genome sequence of the *C. fetus subsp. testudinum* which is primarily isolated from reptile but can cause invasive infection in human was established.	*C*. *fetus* subsp. *testudinum* strain-772	Reptilian food or human–squamate contact was reported as most likely source.	Complete genome sequence established. *C*. *fetus* subsp. *testudinum* from reptiles has zoonotic potential to cause infection in humans.	A patient with chronic kidney disease.	[51]
USA	Positive human infection with new subspecies of genetically distinct variant of *C. fetus*.	*C. fetus* subsp. *testudinum* subsp. *nov*	Reptile. Source reported to be related to traditional asian food or contact with reptile.	*C. fetus* association between reptiles and humans is well illustrated. Infection was related to exposure to foods of reptilian origin or due to human–reptile contact.	Positive cases in nine men of Asian origin, >60 years, with underlying illnesses	[47]
